# Strong Correlation Between Standing Long-Leg Radiographs and CT Scans in Measuring Coronal Knee Alignment

**DOI:** 10.2106/JBJS.23.01092

**Published:** 2024-05-13

**Authors:** Andreas Fontalis, Thomas Luyckx, Thomas Vanspauwen, Robin Moreels, Fabio Mancino, Rhody David Raj, Philip Winnock de Grave, Ricci Plastow, Pierre Putzeys, Fares S. Haddad

**Affiliations:** 1 Department of Trauma and Orthopaedic Surgery, University College Hospital, London, United Kingdom; 2 Division of Surgery and Interventional Science, University College London, London, United Kingdom; 3 Department of Orthopaedic Surgery, AZ Delta Roeselare, Roeselare, Belgium; 4 Hôpitaux Robert Schuman, Luxembourg City, Luxembourg

## Abstract

**Background:**

The objective of this study was to evaluate the correlation in measurements of the lower-limb coronal alignment between long-leg radiographs (LLRs) and computed tomography (CT) scanograms that were made during preoperative planning for robotic-arm-assisted knee arthroplasty. On the basis of published evidence demonstrating a good correlation between these imaging modalities in measuring the lower-limb mechanical axis, we hypothesized that there would be no significant differences between the 2 in the present study.

**Methods:**

This multicenter cohort study across 3 tertiary centers included 300 patients undergoing primary robotic-arm-assisted total knee arthroplasty (TKA) or unicompartmental knee arthroplasty (UKA) for whom LLRs and CT scanograms were available preoperatively. The study involved measuring the medial proximal tibial angle (MPTA), lateral distal femoral angle (LDFA), hip-knee-ankle angle (HKA), joint line obliquity (JLO), joint-line convergence angle (JLCA), and arithmetic HKA (aHKA). The aHKA represents a method for estimating constitutional alignment using angles that are unaffected by joint-space narrowing.

**Results:**

Strong correlations (p < 0.001) between the imaging modalities were found for the HKA (correlation coefficient, 0.912), aHKA (0.883), MPTA (0.820), LDFA (0.871), and JLO (0.778). A weaker correlation was observed for the JLCA in valgus knees as compared with varus knees (Spearman coefficients, 0.412 and 0.518, respectively). Regression models demonstrated that the degree of agreement was associated with the preoperative intra-articular deformity and the positioning of the lower limb during the CT scan (i.e., the lower-limb rotational angle). An initial JLCA within ±5° was associated with higher agreement.

**Conclusions:**

We observed a strong correlation between LLRs and CT scanograms that were made during the preoperative planning stage of robotic-arm-assisted knee arthroplasty, implying that CT scanograms can reliably be utilized to estimate the coronal alignment of the knee, potentially replacing the need for LLRs. Nevertheless, to attain a higher degree of agreement, it is crucial to ensure appropriate radiographic positioning of the lower limb. Additionally, surgeons must remain vigilant regarding potential discrepancies in cases involving substantial deformities.

**Level of Evidence:**

Prognostic Level II. See Instructions for Authors for a complete description of levels of evidence.

Establishing the optimal coronal alignment for patients undergoing knee arthroplasty is a considerable challenge and a contentious topic in contemporary reconstructive knee surgery^1^. Historically, the mechanical hip-knee-ankle angle (HKA) has been the primary metric for quantifying coronal alignment, even though it is influenced by cartilage loss and joint-space narrowing. To date, a double-limb-stance full-leg anteroposterior radiograph of the lower limb is widely utilized to define the coronal alignment of the lower limb. However, with the increasing adoption of computed tomography (CT) scans in preoperative planning, there arises an opportunity to estimate the coronal alignment with use of the CT scanogram. More recently, the arithmetic HKA (aHKA) has emerged as a method that could estimate constitutional coronal alignment in the arthritic population^2^. The aHKA angle utilizes preoperative measurements of the medial proximal tibial angle (MPTA) and lateral distal femoral angle (LDFA) and is characterized by its reliance on osseous landmarks rather than the spatial relationship between the tibia and femur. This approach ensures that the measurements are not influenced by joint-space narrowing, tibiofemoral subluxation, or medial ligamentous laxity^3,4^. Its predictive value has been demonstrated in both arthritic^2^ and nonarthritic populations^4^, as well as in comparisons of the arthritic knee with the unaffected contralateral limb^5^.

The objective of the present study was to evaluate the correlation in measurements of the lower-limb coronal alignment between traditional long-leg radiographs (LLRs) and CT scanograms that were made during the preoperative planning phase. On the basis of published evidence showing a good correlation between these imaging modalities in measuring the lower-limb mechanical axis^6,7^, we hypothesized that no significant differences would be found between the 2 in the present study. Moreover, we aimed to identify whether any variables, such as demographic characteristics, the radiographic positioning of the lower limb, the time between LLRs and CT scans, and the severity of the original deformity, were associated with larger discrepancies between the 2 imaging modalities.

## Materials and Methods

### Study Population

**Fig. 1:**
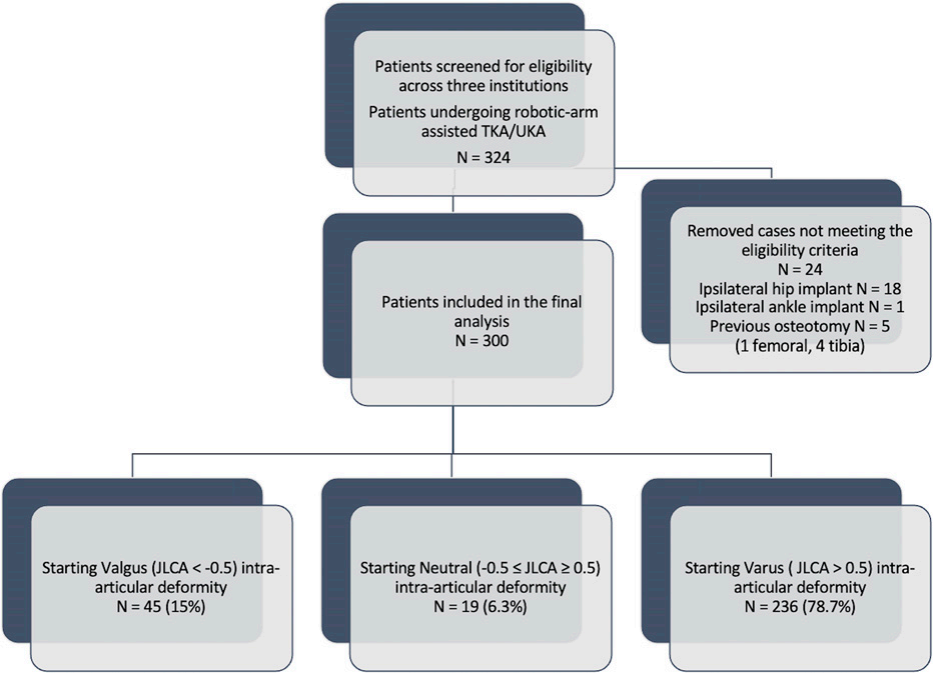
Flowchart depicting the patient selection process.

This multicenter cohort study was conducted across 3 tertiary centers in the United Kingdom, Belgium, and Luxembourg. The study was individually registered and approved at each institution (reference numbers 23058, 230607, and CSS2014-2307) and included 300 adult patients of any age (100 consecutive patients per participating center) undergoing primary robotic-arm-assisted total knee arthroplasty (TKA) or unicompartmental knee arthroplasty (UKA) for symptomatic knee osteoarthritis (Fig. 1). Following screening, participants with available preoperative LLRs and CT scanograms were enrolled into the study. Patients undergoing revision surgery for any cause, patients with an ipsilateral hip or ankle implant, and patients with a previous osteotomy or posttraumatic deformity of the femur or tibia were excluded.

### Imaging Techniques

Two centers utilized conventional radiography to make the LLRs, whereas the third employed the EOS 3D biplanar low-dose imaging system (EOS Imaging). Given our secondary aims to identify variables that had the potential to be associated with greater discrepancies between the 2 imaging modalities and to emulate pragmatic practice when conducting the CT scan, we endeavored to adjudicate the rotation of the lower limb^8^.

**Fig. 2:**
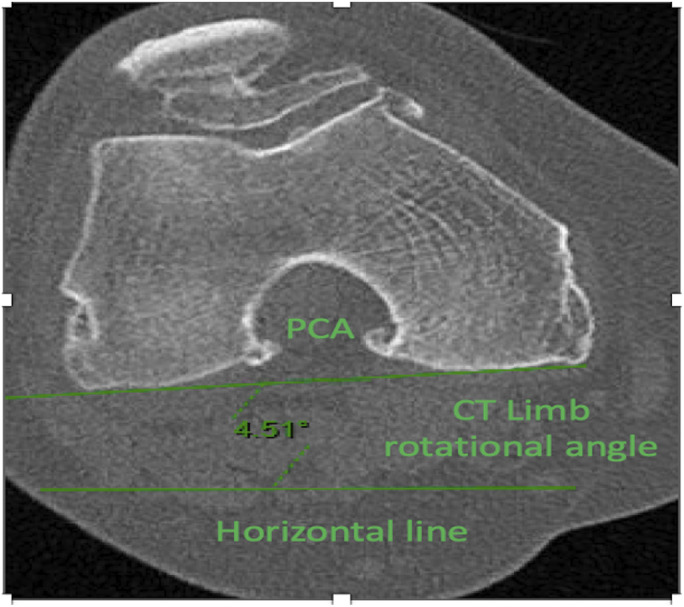
Depiction of the posterior condylar axis (PCA) and CT limb rotational angle (CRA).

To that end, we introduced the concept of the CT limb rotational angle (CRA), which represents the angle subtended by the posterior condylar axis (PCA) and a horizontal line; positive values denote external rotation and negative values denote internal rotation. The PCA was defined as a line connecting the posterior margins of the medial and lateral femoral condyles (Fig. 2). Additionally, we recorded the time interval between when the LLRs and CT scanograms were made (hereafter referred to as the “time between scans”).

### Measurements

A comprehensive range of measurements was utilized to assess coronal knee alignment (Figs. 3-A and 3-B). These measurements included the femoral mechanical axis (fMA), tibial mechanical axis (tMA), LDFA, MPTA, HKA, aHKA, joint line obliquity (JLO), and joint-line convergence angle (JLCA), which was measured on an LLR, a CT scanogram, and a weight-bearing anteroposterior radiograph. The precise definitions and methods of calculation for each measurement are detailed in Table I. To minimize detection bias, the researchers conducting the measurements were blinded to the results of other measurements throughout the study.

**Figs. 3-A and 3-B** Depictions of radiographic measurements.

**Fig. 3-A:**
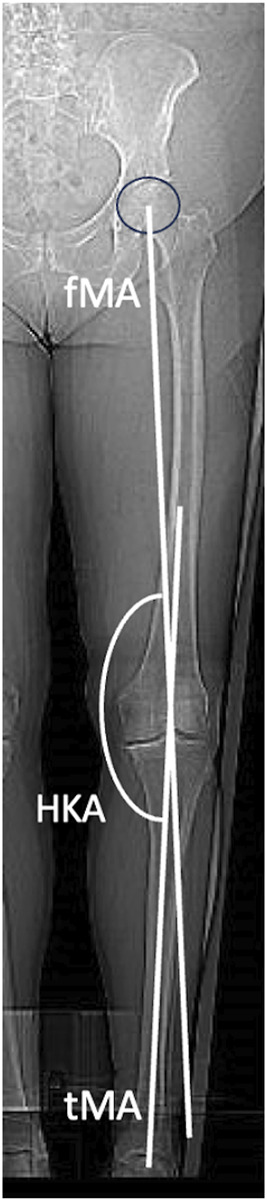
The hip-knee-ankle angle (HKA), femoral mechanical axis (fMA), and tibial mechanical axis (tMA).

**Fig. 3-B:**
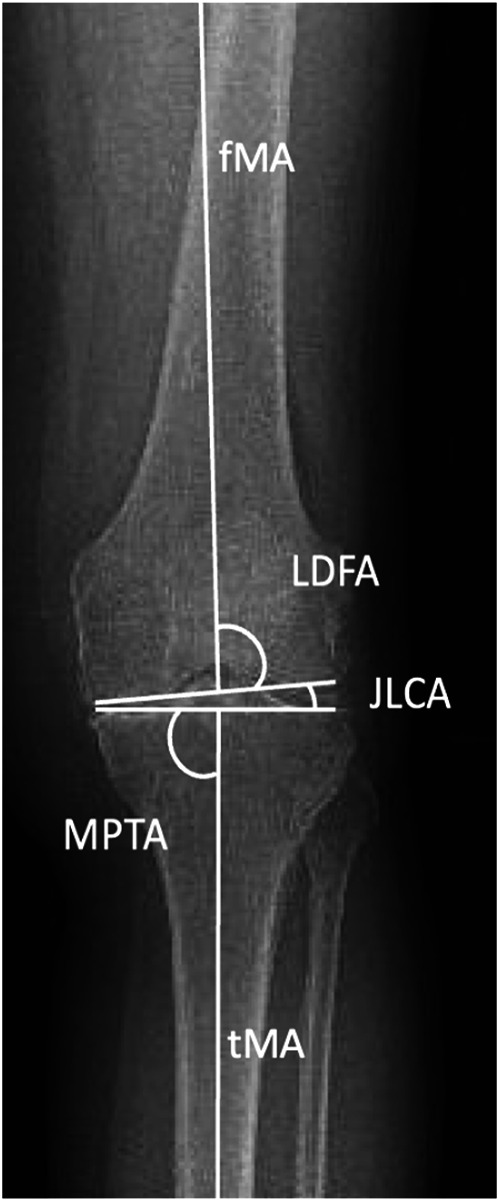
The lateral distal femoral angle (LDFA), medial proximal tibial angle (MPTA), joint-line convergence angle (JLCA), fMA, and tMA.

**TABLE I:** Definitions and Calculation Methods for Knee Alignment Measurements*

Measurement	Definition
Femoral mechanical axis (fMA)	Line from the center of the femoral head to the top of the femoral notch
Tibial mechanical axis (tMA)	Line from the midpoint of the tibia at the knee joint (i.e., the center of the tibial spine) to the center of the tibial plafond at the ankle
Lateral distal femoral angle (LDFA)	Lateral angle subtended by the fMA and a line drawn across the distal femoral articular surface
Medial proximal tibial angle (MPTA)	Medial angle subtended by the tMA and a line drawn across the tibial articular surface at the most distal points
Hip-knee-ankle angle (HKA)	Medial angle between the fMA and the tMA
Arithmetic hip-knee-ankle angle (aHKA)	Calculated by subtracting the LDFA from the MPTA
Joint line obliquity (JLO)	Calculated by adding the LDFA and the MPTA
Joint-line convergence angle (JLCA)	Measured on LLR, CT scanogram, and weight-bearing anteroposterior radiograph to gauge intra-articular deformity

* LLR = long-leg radiographs, CT = computed tomography.

### Objectives

The primary objective of this study was to evaluate the correlation between LLRs and CT scanograms in measuring the coronal alignment of the lower limb. In addition, we utilized regression models to identify variables that were associated with greater discrepancies in measurements between the 2 imaging modalities and further explored the agreement between the imaging modalities at various levels of those variables. Sensitivity analyses were also conducted to determine if the agreement between the LLRs and CT scanograms differed when these modalities were utilized to examine patients with medial or lateral osteoarthritis.

### Power Analysis and Sample Size Calculation

To determine the required sample size, we conducted a priori power analyses. Our aim was to ensure sufficient statistical power to identify even a marginal effect size and correlation between the 2 imaging modalities. To achieve a statistical power of 0.9 for detecting a correlation coefficient of 0.2 at a significance level of 0.05, a minimum of 259 paired observations were necessary^9,10^. To account for potential data loss, outliers, or unforeseen circumstances that could impact our analysis, our study included a total of 300 paired observations.

### Statistical Analysis

The correlation between the 2 imaging modalities was quantified with use of either the Pearson or Spearman correlation coefficient, depending on the data distribution. Furthermore, Bland-Altman plots were utilized to assess the agreement between the 2 methods, to visualize outliers, and to detect potential systematic bias. These plots illustrate the 95% limits of agreement, determined by the mean difference ± 1.96 standard deviations (SDs), and indicate the expected range of agreement within which the measurements from both methods were anticipated to fall. To evaluate interobserver agreement, intraclass correlation coefficients were calculated with use of a 2-way mixed-effects model with absolute agreement. To identify factors contributing to larger discrepancies between the 2 imaging modalities, hierarchical multivariable linear regression models were employed. These models adjusted for demographic factors (age and gender), time between scans, the CRA, and the magnitude of the original deformity (the preoperative JLCA angle). Additionally, to assess the agreement between LLRs and CT scanograms across different levels of variables that were associated with larger discrepancies in the multivariable model, we calculated and plotted the mean difference between the 2 modalities for each radiographic parameter at various levels of the variables. We implemented a systematic approach starting at a threshold of 1 and proceeding with sequential 1-unit increases. This approach allowed a granular analysis of how agreement changed with incremental differences in a variable. For each threshold, a subset of the cohort was selected on the basis of observations that fell within the positive and negative values of the threshold. The mean difference between the imaging modalities was then calculated for these subgroups. A 2-tailed p value of <0.05 was set as the level of significance.

## Results

The mean age of our study population was 69 years (range, 40 to 87 years), and female patients constituted 41% of the total cohort (Table II). Most (83.7%) of the patients underwent TKA. The remaining patients underwent UKA, which involved the medial compartment in 95.9% of those patients. The median JLCA based on an anteroposterior radiograph of the arthritic knee was 3.4° (quartile 1 to quartile 3, 1° to 5°). The median CRA was 5° (quartile 1 to quartile 3, 1.2° to 10.5°).

**TABLE II:** Baseline Characteristics and Demographics of the Study Cohort (N = 300)*

Variable	Patients Undergoing Robotic-Arm-Assisted TKA or UKA
Age *(yr)*	69 (40-87)
Gender *(no. [%] of patients)*	
** **Female	122 (40.7)
** **Male	178 (59.3)
Laterality *(no. [%] of patients)*	
** **Right	155 (51.7)
** **Left	145 (48.3)
Procedure *(no. [%] of patients)*	
** **TKA	251 (83.7)
** **UKA	49 (16.3)
Medial	47 (95.9)
Lateral	2 (4.1)
JLCA† *(deg)*	3.4 (1, 5)
Preoperative intra-articular deformity *(no. [%] of patients)*	
** **Valgus (JLCA < −0.5°)	45 (15)
** **Neutral (−0.5° ≤ JLCA ≤ 0.5°)	19 (6.3)
** **Varus (JLCA > 0.5°)	236 (78.7)
CRA *(deg)*	5 (1.2, 10.5)
KL grade, medial compartment *(no. [%] of patients)*	
** **0	0
** **1	1 (0.3)
** **2	38 (12.7)
** **3	89 (29.7)
** **4	172 (57.3)
KL grade, lateral compartment *(no. [%] of patients)*	
** **0	1 (0.3)
** **1	22 (7.3)
** **2	118 (39.3)
** **3	120 (40)
** **4	39 (13)
Time between LLRs and CT scanogram *(days)*	0 (0, 40)

* Categorical variables are presented as the absolute number, with the percentage in parentheses. Continuous variables are presented as the median, with quartiles 1 and 3 in parentheses, except for age, which is given as the mean, with the range in parentheses. KL = Kellgren-Lawrence system for the classification of osteoarthritis.

† Based on an anteroposterior knee radiograph.

### Correlational Analyses

Table III displays the radiographic measurements obtained from the 2 imaging modalities. The median HKA was 174.6° (quartile 1 to quartile 3, 171.8° to 179.1°) on LLRs and 174.8° (quartile 1 to quartile 3, 171.6° to 179°) on CT scanograms, with a mean difference of 0.37°. The mean aHKA was −1° on LLRs and −2.3° on CT scanograms, with a mean difference of 1.3°. The mean JLO was 175.20° on LLRs and 174.45° on CT scanograms, with a mean difference of 0.75°.

**TABLE III:** Comparison of Radiographic Measurements Between LLRs and CT Scanograms

Variable	LLR* *(deg)*	CT Scanogram* *(deg)*	Mean Difference ± SD *(deg)*	Correlation Coeff.	P Value
HKA	174.6 (171.8, 179.1)	174.8 (171.6, 179)	0.37 ± 2.6	0.912†	<0.001
LDFA	88.13 ± 3	88.39 ± 3.2	−0.26 ± 1.6	0.871‡	<0.001
MPTA	87.1 ± 3.3	86 ± 3.7	1.1 ± 2.1	0.820‡	<0.001
aHKA	−1 ± 5.1	−2.3 ± 5.3	1.3 ± 2.5	0.883‡	<0.001
JLO	175.20 ± 3.9	174.45 ± 4.4	0.75 ± 2.7	0.778‡	<0.001
JLCA	3.3 (0.8, 5.3)	2 (0.3, 3.2)	1.1 ± 2.3	0.690†	<0.001

* Variables are presented as the mean ± SD or as the median with quartiles 1 and 3 in parentheses.

† Spearman correlation coefficient.

‡ Pearson correlation coefficient.

The intraclass correlation coefficient ranged from 0.952 to 0.988 for the different variables, suggesting excellent interobserver agreement. Table III demonstrates the correlation between LLRs and CT scanograms across various metrics utilized to characterize the lower-limb constitutional alignment. The results collectively suggested a strong and significant correlation (p < 0.001) between the 2 imaging modalities for all studied parameters. The JLCA was the metric with the weakest correlation, but it still demonstrated substantial agreement (Spearman correlation, 0.690).

**Fig. 4:**
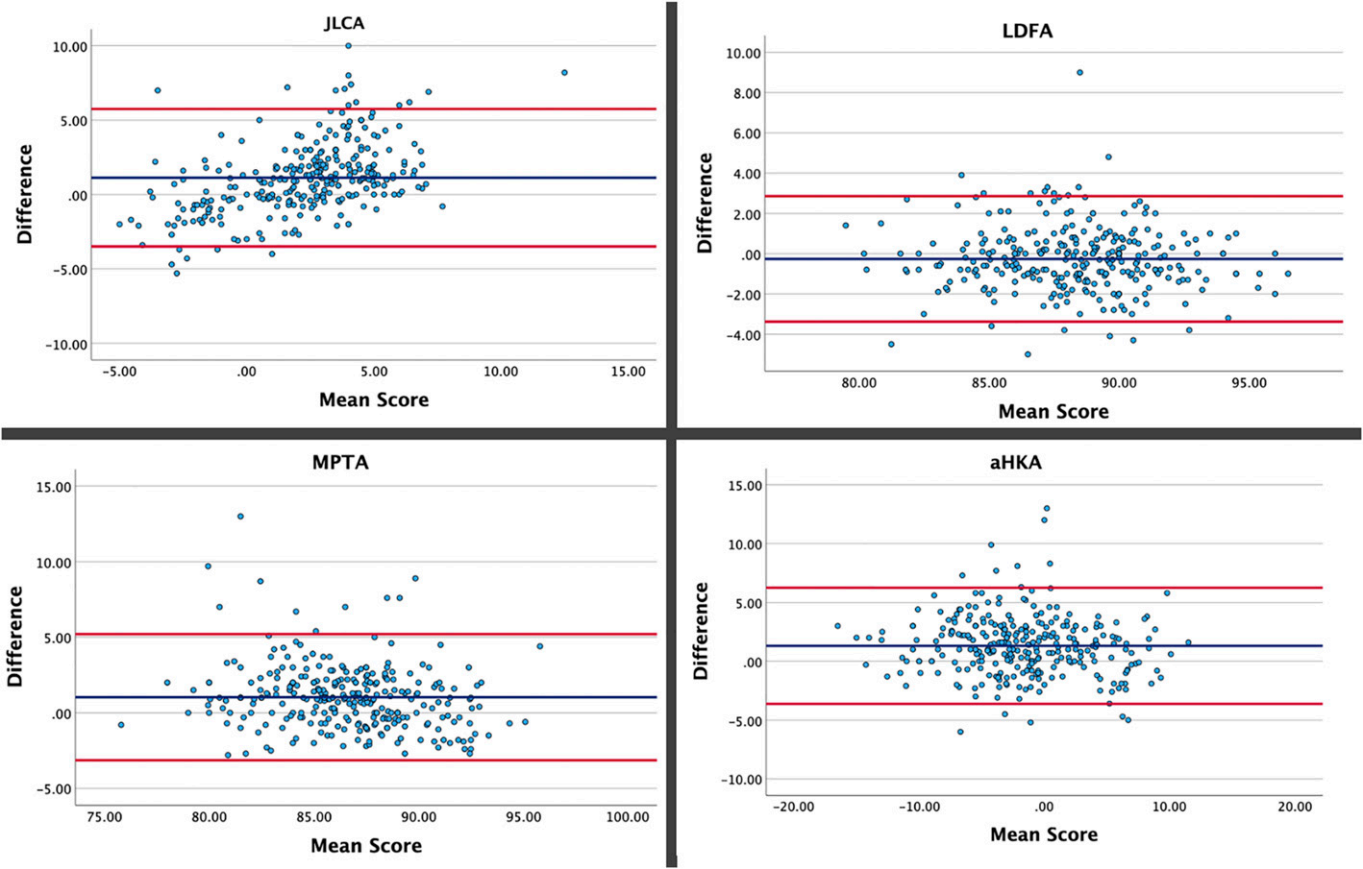
Bland-Altman plots for the JLCA, LDFA, MPTA, and aHKA. The solid red lines represent the 95% limits of agreement (±1.96 SDs), and the solid blue line represents the mean.

Figure 4 illustrates the Bland-Altman plots for the different variables. The percentage of observations that fell within the limits of agreement was 96.6% for the MPTA, 95.7% for the aHKA, 94.3% for the LDFA, 94.6% for the HKA, and 94% for the JLCA.

### Sensitivity Analysis

A subgroup analysis was conducted to evaluate the impact of the initial deformity on the agreement between the 2 imaging techniques. Separate correlational analyses were performed for patients with an initial varus deformity (i.e., a JLCA of >0° on a weight-bearing anteroposterior radiograph) and those with an initial valgus deformity (i.e., a JLCA of <0° on a weight-bearing anteroposterior radiograph). The findings suggest that the correlation for the aHKA was comparable between valgus and varus knees (correlation coefficients of 0.855 and 0.844, respectively) but that the correlation for most of the other measured variables was marginally weaker for valgus knees, with correlation coefficients ranging from 0.412 for the JLCA to 0.845 for the LDFA (Table IV).

**TABLE IV:** Correlation of Radiographic Measurements Between LLRs and CT Scanograms of Knees with a Preoperative Varus or Valgus Intra-Articular Deformity

Variable	Preoperative Varus Intra-Articular Deformity*				Preoperative Valgus Intra-Articular Deformity†			
	LLR‡ *(deg)*	CT Scanogram‡ *(deg)*	Correlation Coeff.	P Value	LLR‡ *(deg)*	CT Scanogram‡ *(deg)*	Correlation Coeff.	P Value
HKA	173.8 (171, 176.7)	173.9 (170.9, 176.5)	0.873§	<0.001	186.65 (181.92, 190.2)	184.3 (181.4, 187.1)	0.839§	<0.001
LDFA	88.76 ± 2.64	89 ± 2.89	0.862#	<0.001	85.17 ± 3.1	85.4 ± 2.7	0.845#	<0.001
MPTA	86.50 (84.8, 88.3)	85.4 (83.1, 87.5)	0.826§	<0.001	89.60 ± 2.7	89.2 ± 2.7	0.797#	<0.001
aHKA	−2.2 ± 4.3	−3.6 ± 4.6	0.844#	<0.001	4.4 ± 4.3	3.7 ± 4.1	0.855#	<0.001
JLO	175.3 ± 4	174.4 ± 4.5	0.825#	<0.001	174.80 ± 3.7	174.6 ± 3.6	0.769#	<0.001
JLCA	4 (2.4, 5.7)	2.25 (1.2, 3.6)	0.518§	<0.001	−2.1 (−3.47, −0.72)	−1.35 (−2.07, −0.52)	0.412§	0.002

* A JLCA of >0° on a weight-bearing radiograph.

† A JLCA of <0° on a weight-bearing radiograph.

‡ Variables are presented as the mean ± SD or as the median with quartiles 1 and 3 in parentheses.

§ Spearman correlation coefficient.

# Pearson correlation coefficient.

### Identification of Parameters Associated with Larger Discrepancies

After adjustments were made for age, gender, the time between scans, and the CRA, linear regression models showed that the magnitude of the original deformity (i.e., the absolute value of the JLCA on a weight-bearing anteroposterior radiograph) was significantly associated with the absolute difference in JLCA measurements between LLRs and CT scanograms (β = 0.220; 95% confidence interval [CI], 0.132 to 0.308; p < 0.001).

After adjusting for age, gender, laterality, preoperative deformity (JLCA), and the time between scans, the absolute value of the CRA was associated with the discrepancy in MPTA measurements between LLRs and CT scanograms (β = 0.064; 95% CI, 0.037 to 0.091; p < 0.001). An association between the absolute value of the CRA and the error in measurements was also noted for the HKA (β = 0.049; 95% CI, 0.019 to 0.079; p = 0.002), JLO (β = 0.094; 95% CI, 0.061 to 0.128; p < 0.001), and aHKA (β = 0.038; 95% CI, 0.007 to 0.069; p = 0.017). No significant association was found for the LDFA (β = 0.14; 95% CI, −0.004 to 0.033; p = 0.132) or JLCA (β = −0.005; 95%, CI −0.034 to 0.023; p = 0.715).

### Evaluating Agreement at Different JLCA and CRA Thresholds

**Fig. 5:**
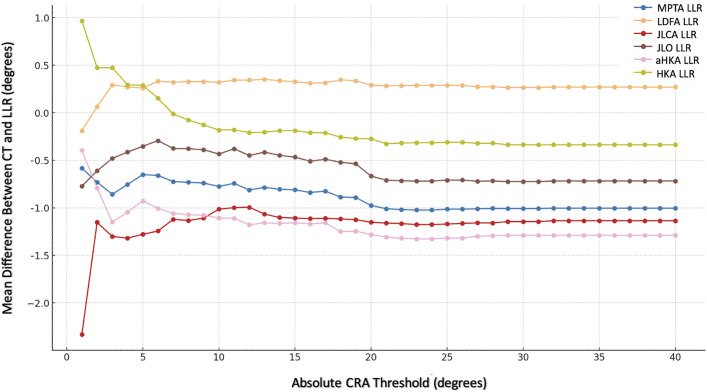
Graph illustrating the mean difference in measurements between the 2 imaging modalities at different thresholds of the CRA. For each CRA threshold on the x axis, the subset of the cohort with observations that were within the positive and negative values of the threshold is included. The mean difference between the 2 imaging modalities for these observations is displayed on the y axis.

**Fig. 6:**
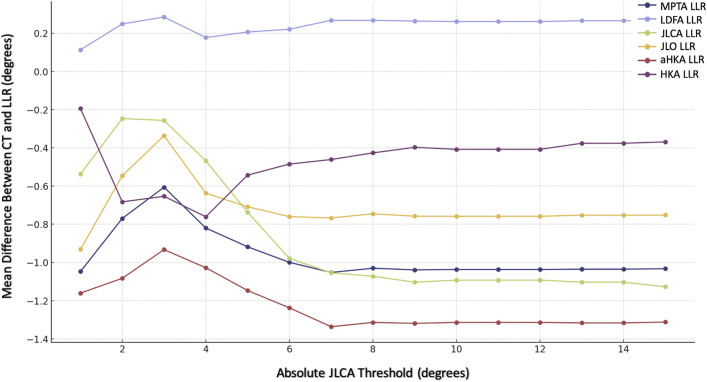
Graph illustrating the mean difference in measurements between the 2 imaging modalities at different thresholds of the preoperative intra-articular deformity (i.e., the JLCA on a weight-bearing anteroposterior radiograph). For each JLCA threshold on the x axis, the subset of the cohort with observations that were within the positive and negative values of the threshold is included. The mean difference between the 2 imaging modalities for these observations is displayed on the y axis.

To evaluate agreement between the imaging modalities at different levels, we calculated and plotted the mean difference for each radiographic parameter at various thresholds of the CRA and the JLCA (Tables V and VI, Figs. 5 and 6).

**TABLE V:** Mean Differences in Radiographic Measurements Between CT Scanograms and LLRs at Different Thresholds of the Absolute CRA*

CRA Threshold	MPTA	LDFA	JLCA	JLO	aHKA	HKA
1	−0.584	−0.188	−2.332	−0.772	−0.396	0.964
2	−0.730	0.063	−1.153	−0.611	−0.793	0.472
3	−0.858	0.291	−1.301	−0.480	−1.149	0.473
4	−0.753	0.271	−1.318	−0.4140	−1.045	0.292
5	−0.651	0.259	−1.279	−0.353	−0.926	0.289
6	−0.659	0.331	−1.244	−0.294	−1.006	0.152
7	−0.724	0.321	−1.121	−0.376	−1.059	−0.013
8	−0.731	0.327	−1.134	−0.379	−1.071	−0.076
9	−0.740	0.327	−1.108	−0.389	−1.079	−0.128
10	−0.776	0.319	−1.014	−0.434	−1.106	−0.182
11	−0.743	0.342	−0.998	−0.379	−1.110	−0.179
12	−0.812	0.342	−0.995	−0.449	−1.178	−0.209
13	−0.785	0.350	−1.065	−0.415	−1.158	−0.205
14	−0.805	0.336	−1.102	−0.448	−1.163	−0.189
15	−0.811	0.326	−1.108	−0.467	−1.158	−0.188
16	−0.839	0.310	−1.113	−0.510	−1.169	−0.214
17	−0.824	0.315	−1.110	−0.490	−1.158	−0.211
18	−0.886	0.346	−1.116	−0.521	−1.252	−0.257
19	−0.891	0.335	−1.124	−0.537	−1.244	−0.271
20	−0.975	0.290	−1.152	−0.666	−1.281	−0.274
21	−1.009	0.282	−1.160	−0.709	−1.308	−0.326
22	−1.018	0.285	−1.165	−0.715	−1.319	−0.318
23	−1.023	0.287	−1.176	−0.719	−1.327	−0.316
24	−1.023	0.287	−1.176	−0.719	−1.327	−0.316
25	−1.014	0.288	−1.170	−0.709	−1.318	−0.311
26	−1.014	0.288	−1.164	−0.708	−1.318	−0.311
27	−1.010	0.272	−1.158	−0.720	−1.299	−0.322
28	−1.006	0.271	−1.159	−0.717	−1.293	−0.320
29	−1.007	0.265	−1.145	−0.725	−1.289	−0.337
30	−1.007	0.265	−1.145	−0.725	−1.289	−0.337
31	−1.007	0.265	−1.145	−0.725	−1.289	−0.337
32	−1.004	0.269	−1.136	−0.717	−1.289	−0.337
33	−1.004	0.269	−1.136	−0.717	−1.289	−0.337
34	−1.004	0.269	−1.136	−0.717	−1.289	−0.337
35	−1.004	0.269	−1.136	−0.717	−1.289	−0.337
36	−1.004	0.269	−1.136	−0.717	−1.289	−0.337
37	−1.004	0.269	−1.136	−0.717	−1.289	−0.337
38	−1.004	0.269	−1.136	−0.717	−1.289	−0.337
39	−1.004	0.269	−1.136	−0.717	−1.289	−0.337
40	−1.004	0.269	−1.136	−0.717	−1.289	−0.337
41	−1.004	0.269	−1.136	−0.717	−1.289	−0.337
42	−1.033	0.263	−1.127	−0.752	−1.312	−0.369

* All values are given as degrees. For each threshold, the subset of the cohort with observations that were within the positive and negative values of the threshold is included and the mean difference is presented.

**TABLE VI:** Mean Differences in Radiographic Measurements Between CT Scanograms and LLRs at Different Thresholds of the Preoperative Intra-Articular Deformity*

Intra-Articular Deformity Threshold	MPTA	LDFA	JLCA	JLO	aHKA	HKA
1	−1.047	0.113	−0.537	−0.932	−1.160	−0.195
2	−0.770	0.249	−0.247	−0.546	−1.083	−0.683
3	−0.606	0.285	−0.256	−0.336	−0.933	−0.654
4	−0.820	0.177	−0.468	−0.638	−1.027	−0.761
5	−0.918	0.206	−0.737	−0.709	−1.147	−0.543
6	−0.999	0.221	−0.979	−0.760	−1.237	−0.485
7	−1.052	0.267	−1.054	−0.767	−1.337	−0.461
8	−1.030	0.267	−1.072	−0.745	−1.314	−0.426
9	−1.039	0.264	−1.103	−0.758	−1.319	−0.397
10	−1.037	0.261	−1.093	−0.758	−1.314	−0.408
11	−1.037	0.261	−1.093	−0.758	−1.314	−0.408
12	−1.037	0.261	−1.093	−0.758	−1.314	−0.408
13	−1.035	0.265	−1.103	−0.753	−1.317	−0.376
14	−1.035	0.265	−1.103	−0.753	−1.317	−0.376
15	−1.033	0.263	−1.127	−0.752	−1.312	−0.369

* All values are given as degrees. The preoperative intra-articular deformity was measured as the absolute value of the JLCA on a weight-bearing radiograph. For each threshold, the subset of the cohort with observations that were within the positive and negative values of the threshold is included and the mean difference is presented.

A preoperative intra-articular deformity (JLCA) within ±5° was associated with higher agreement between the 2 modalities (mean absolute differences of <1° for the MPTA, LDFA, JLCA, and JLO; Fig. 6).

With respect to the CRA, higher agreement (a mean absolute difference of <1°) between the 2 modalities was observed for the MPTA when the CRA was within ±20°; for the aHKA, when the CRA was within ±6°; and for the JLCA, when the CRA was within ±12°.

## Discussion

Our findings underlined a robust correlation between LLRs and CT scanograms across all parameters that were utilized for the estimation of coronal alignment. The JLCA demonstrated the weakest correlation (Spearman coefficient, 0.69), but the degree of agreement was still substantial, with a mean difference of 1.1°. This mean difference falls within the discrepancy range of up to 1.5° between the measurement results, which prior research has shown is acceptable for demonstrating equivalence^5,11^. Previous studies have established the utility of the aHKA in estimating the constitutional alignment^2,5^. The present study is distinctive as it explored the correlation between LLRs and CT scanograms with respect to measurements that are integral to the computation of the aHKA.

The first variable that was identified as affecting the agreement between LLRs and CT scanograms was the degree of intra-articular deformity, as represented by the JLCA. The second was the positioning of the lower limb during the CT scanogram, as represented by the CRA. Our data suggested a higher degree of agreement when the initial JLCA, which was based on a weight-bearing anteroposterior knee radiograph, remained within a range of ±5°. Importantly, even when larger thresholds were applied, the correlation between the 2 imaging techniques remained substantial and clinically acceptable and the maximum mean difference for any of the variables did not exceed 1.5°^5,11^.

Given that CT scanograms eliminate weight-bearing conditions and thereby the ground reaction force, potential ligamentous imbalance^12^ or cartilage loss^13^ may not be accurately reflected on such images. Consequently, it is crucial for knee arthroplasty surgeons to be cognizant of the level of agreement between the LLR and the CT scanogram when estimating the coronal knee alignment in the arthritic population (aHKA). The aforementioned limitation could account for the weaker correlation in measurements of the JLCA of valgus knees that was observed in the present study. Specifically, valgus knees may exhibit more substantial cartilage loss in the tibial plateau and greater laxity of the medial collateral ligament, leading to a diminished correlation between LLRs and CT scanograms with respect to JLCA measurements.

Several published studies have tried to examine the agreement between weight-bearing and non-weight-bearing imaging modalities^3,6,7,14-19^. However, these studies primarily compared intraoperative measurements derived from imageless computer-assisted surgery (CAS) or robotic systems with measurements derived from LLRs and focused on the correlation between the imaging modalities in terms of the mechanical alignment (HKA) rather than the constitutional alignment (aHKA) or the parameters involved in calculating the aHKA. Sabharwal and Zhao found that a JLCA of >3° and a mechanical axis deviation of >2 cm were each associated with a significantly greater discrepancy between supine fluoroscopy images and standing full-length radiographs^19^. Babazadeh et al. observed a robust correlation between LLR and CT scan measurements but noted limited agreement when comparing either of these modalities with CAS measurements^7^. Both Willcox et al.^18^ and Meijer et al.^17^ highlighted inconsistencies between intraoperative CAS measurements and LLR measurements, with the latter frequently indicating a more substantial deformity. In a sample of 200 patients, Shetty et al. explored the influence of sagittal deformity on coronal mechanical alignment measured with use of either LLRs or intraoperative navigation^16^. The authors reported a larger discrepancy in the measurements when the fixed flexion deformity surpassed 10°. However, their research focused exclusively on the correlation.

Studies that are the most akin to ours have compared CT scanograms with LLRs in measuring the mechanical axis. One such study by Gbejuade et al. found good agreement between the imaging modalities, albeit with reduced agreement in cases of more pronounced deformities^6^. However, Gbejuade et al. examined the correlation solely in terms of the mechanical axis and did not consider the parameters utilized for the aHKA computation. Holme et al. evaluated the agreement between LLRs and CT scanograms in determining mechanical and anatomical axes in patients undergoing UKA^15^. The authors reported that both modalities were reliable but that significant and clinically relevant differences were evident. However, the sample size was small (40 knees, 23 of which were post-UKA), and the study did not assess measurements of the constitutional alignment and aHKA.

Another important factor is the irradiation associated with the studied imaging modalities. Full LLRs entail significant radiation exposure and may present challenges for patients with weight-bearing-related difficulties or patients who use wheelchairs^6^. The estimated radiation dose for a CT scanogram ranges from 0.1 to 0.2 mSv^6,20^, whereas the average dose for a conventional LLR is 0.7 mSv^20^. However, it should be noted that there is considerable variation in the reported radiation doses in the literature, depending on the specific CT protocol that was utilized^6,21,22^.

The present study features several strengths, including a large sample size and multicentricity. Additional strengths include the consideration of variables that could potentially have confounded our results, including the time interval between the scans, the lower-limb rotation during the CT scan, and the JLCA. Prior research has indicated that malrotation of the lower limb is commonly present when making LLRs, often leading to measurement errors^23^. To that end, we introduced the concept of the CRA, which was aimed at evaluating lower-limb rotation. Moreover, we sought to identify parameters that were associated with a larger discrepancy and to evaluate agreement at different levels of these parameters. Prior research has reported that a discrepancy of up to 1.5° in the measurement results is acceptable for demonstrating equivalence^5,11^. This threshold falls within the permissible error margin for radiographic evaluations as well as optical computer-assisted navigation systems^11,24,25^.

The main limitations of the present study pertain to the observational study design and exclusion criteria. Patients with a previous osteotomy, posttraumatic deformity, or an ipsilateral hip or ankle implant were not included. Consequently, extrapolation of our findings to these patient groups may be limited.

In conclusion, our study demonstrated a robust correlation between LLRs and CT scanograms utilized in preoperative planning for robotic-arm-assisted knee arthroplasty. This finding suggests that CT scanograms could reliably replace LLRs for estimating the coronal alignment, offering benefits such as reduced irradiation and resource use. However, to attain a higher degree of agreement, particular attention should be paid by the CT technicians and radiographers in ensuring appropriate patient positioning, and arthroplasty surgeons should remain vigilant in cases involving pronounced deformities. Despite these considerations, our study revealed a significant and clinically acceptable level of agreement between the 2 imaging modalities.
